# Traditional Antipyretic Medicinal Plants Used in Yasothon Province, Northeastern Thailand

**DOI:** 10.3390/plants15152373

**Published:** 2026-08-02

**Authors:** Tammanoon Jitpromma, Piyaporn Saensouk, Santi Watthana, Pathomthat Srisuk, Khwanjai Thanakornjuk, Surapon Saensouk

**Affiliations:** 1 Diversity of Family Zingiberaceae and Vascular Plant for Its Applications Research Unit, Mahasarakham University, Maha Sarakham 44150, Thailand; jitpromma.t@gmail.com (T.J.); pcornukaempferia@yahoo.com (P.S.); 2 Walai Rukhavej Botanical Research Institute, Mahasarakham University, Maha Sarakham 44150, Thailand; 3 Department of Biology, Faculty of Science, Mahasarakham University, Maha Sarakham 44150, Thailand; 4 School of Biology, Institute of Science, Suranaree University of Technology, Nakhon Ratchasima 30000, Thailand; santibio@sut.ac.th; 5 Department of Pharmaceutical Technology, Faculty of Pharmaceutical Sciences, Khon Kaen University, Khon Kaen 40002, Thailand; spatho@kku.ac.th; 6 Tha Uthen Hospital, 23/23 moo.6 Nontan Thauthen, Nakhonphanom 48120, Thailand; tkjai2521@yahoo.co.th

**Keywords:** ethnomedicine, fever, herbal plants, medicinal plants, traditional healing practices, traditional knowledge, Yasothon Province

## Abstract

Fever is one of the most common health conditions traditionally treated with medicinal plants in rural communities, yet comprehensive documentation of antipyretic ethnomedicinal knowledge in northeastern Thailand remains limited. This study presents a focused ethnobotanical analysis of medicinal plants used for fever treatment in Yasothon Province, northeastern Thailand, and assesses informant agreement regarding their antipyretic use using the fidelity level (FL). Ethnomedicinal data were collected through semi-structured interviews, participant observation, and guided field walks with 108 informants from nine districts between April 2024 and February 2026, and records related to fever treatment were extracted for detailed analysis. Voucher specimens were collected and taxonomically verified. A total of 129 medicinal plant species belonging to 108 genera and 46 families were recorded. Fabaceae was the most represented family, while native species predominated (83.72%). Trees constituted the largest growth form (45.74%), and wild plants accounted for 62.02% of all recorded species. Roots were the most frequently used plant part (39.53%), and decoction was the principal preparation method (93.02%). Twenty-five species exhibited an FL value of 100%. These findings highlight the richness of traditional antipyretic knowledge in Yasothon Province and provide a useful baseline for the preservation of biocultural heritage and for future ethnopharmacological research on medicinal plants used in fever treatment.

## 1. Introduction

Fever is one of the most prevalent clinical manifestations associated with infectious and inflammatory diseases and remains a major public health concern worldwide [1]. Although fever is a physiological response that enhances host defense mechanisms, persistent or excessive elevation of body temperature can increase metabolic demand and lead to severe complications, particularly in children, older adults, and immunocompromised individuals [2]. Consequently, medicinal plants with antipyretic properties have long been incorporated into traditional healthcare systems as accessible, affordable, and culturally accepted therapeutic resources [3].

Plant biodiversity is a fundamental component of global ecosystem functioning and has recently received considerable scientific attention in relation to the diversity of plant secondary metabolites and their potential health-promoting properties. These bioactive compounds are increasingly studied due to growing global interest in natural products, functional foods, and plant-derived therapeutic agents [4].

Traditional medicinal knowledge represents a dynamic body of empirical observations accumulated through generations of interactions between humans and their surrounding environments [5]. Ethnobotanical investigations have demonstrated that medicinal plants continue to play an essential role in primary healthcare, especially in rural communities where local biodiversity provides readily available therapeutic resources [6]. Beyond their cultural significance, medicinal plants constitute valuable sources of bioactive compounds, including flavonoids, alkaloids, terpenoids, phenolics, and glycosides, many of which exhibit antipyretic, anti-inflammatory, antioxidant, and antimicrobial activities [7,8]. Therefore, documenting traditional medicinal knowledge not only preserves intangible cultural heritage but also provides a scientific foundation for pharmacological research and sustainable utilization of plant resources [9].

Particular scientific interest has recently been directed toward rare and underutilized medicinal plant species from geographically and climatically diverse regions that remain insufficiently explored. These species may possess unique phytochemical profiles and biological activities as a result of their specific environmental and climatic growth conditions. Such biodiversity hotspots are especially important for contemporary biotechnological research, including plant conservation, propagation, metabolic profiling, and the sustainable utilization of valuable plant genetic resources [10].

Thailand possesses remarkable floristic diversity and a rich tradition of herbal medicine. Numerous ethnobotanical studies conducted throughout the country have documented medicinal plant utilization for various ailments; however, investigations specifically focusing on antipyretic plants remain scarce [11]. In Yasothon Province, recent ethnobotanical studies in Mueang District have substantially improved our understanding of local plant use. An ethnobotanical survey of edible plants documented only seven species used for fever treatment among 170 edible taxa, indicating that antipyretic applications represent only a small component of a broader food-use system [12]. Another study reported merely three medicinal species employed for fever management within a wider ethnomedicinal inventory [13]. Likewise, investigations of useful plants and wild edible plants identified only eight and three antipyretic species, respectively, because their primary objectives emphasized overall plant diversity rather than fever-specific traditional knowledge [14,15]. Collectively, these studies demonstrate that although medicinal plants for fever are recognized within local communities, their diversity, cultural importance, preparation methods, therapeutic consensus, and ethnomedicinal significance have never been systematically investigated.

The limited number of antipyretic species reported in previous studies does not necessarily reflect low medicinal diversity but rather highlights the absence of dedicated ethnobotanical research on fever treatment. Traditional healers and local communities frequently employ multiple plant species, different plant parts, and diverse preparation methods according to symptom severity, patient characteristics, and inherited cultural practices [16]. Without comprehensive documentation, this specialized knowledge faces increasing risks of erosion due to modernization, socioeconomic transformation, and declining intergenerational knowledge transmission [17,18].

A focused ethnobotanical investigation of antipyretic medicinal plants is therefore essential for preserving local biocultural heritage while improving our understanding of traditional healthcare practices and the diversity of plant resources used in fever management [19]. Documenting local knowledge contributes not only to the conservation of intangible cultural heritage but also provides valuable baseline information for future phytochemical, pharmacological, and conservation studies [20]. Traditional remedies that have been maintained through generations represent an important reservoir of empirical knowledge and may serve as promising sources for the discovery of novel bioactive compounds and the development of sustainable plant-based healthcare strategies [21].

Accordingly, the present study aims to comprehensively document the traditional antipyretic medicinal plants used in Yasothon Province, northeastern Thailand. Specifically, this study (i) inventories the plant species used for fever treatment; (ii) documents their distribution, growth forms, resource types, plant parts used, preparation methods, and conditions of use; (iii) evaluates the degree of informant consensus using fidelity level (FL); and (iv) provides baseline information for the preservation of traditional knowledge, biodiversity conservation, and future ethnopharmacological and phytochemical research. By focusing exclusively on antipyretic medicinal plants, this study expands the existing ethnobotanical knowledge of Yasothon Province and contributes a comprehensive reference for traditional fever remedies in northeastern Thailand.

## 2. Results

### 2.1. Diversity of Antipyretic Medicinal Plants

**Figure 1:**
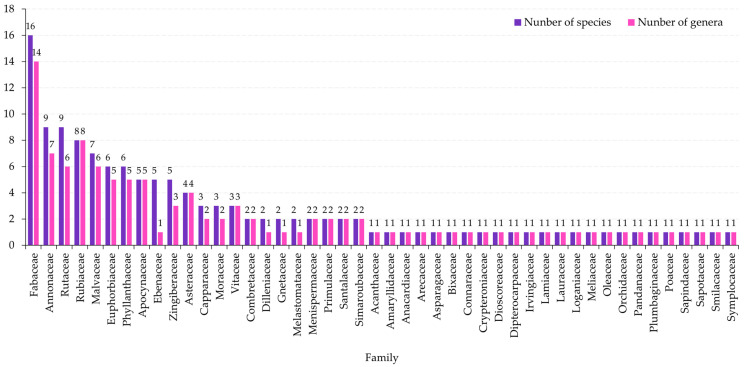
Taxonomic diversity of antipyretic medicinal plants recorded in Yasothon Province, northeastern Thailand, showing the number of species and genera represented in each plant family.

A total of 129 antipyretic medicinal plant species, belonging to 108 genera and 46 families, were documented in Yasothon Province (Table 1, Figure 1). The recorded flora exhibited considerable taxonomic diversity, with most families represented by only one or a few species.

Based on species richness, Fabaceae was the dominant family, comprising 16 species, followed by Annonaceae and Rutaceae, each represented by nine species. Rubiaceae ranked fourth with eight species, while Malvaceae contained seven species. Euphorbiaceae and Phyllanthaceae each contributed six species, whereas Apocynaceae, Ebenaceae, and Zingiberaceae were represented by five species each. Asteraceae included four species, and Capparaceae, Moraceae, and Vitaceae each contained three species. Eight families were represented by two species, while the remaining 24 families were each represented by a single species.

**Figure 2:**
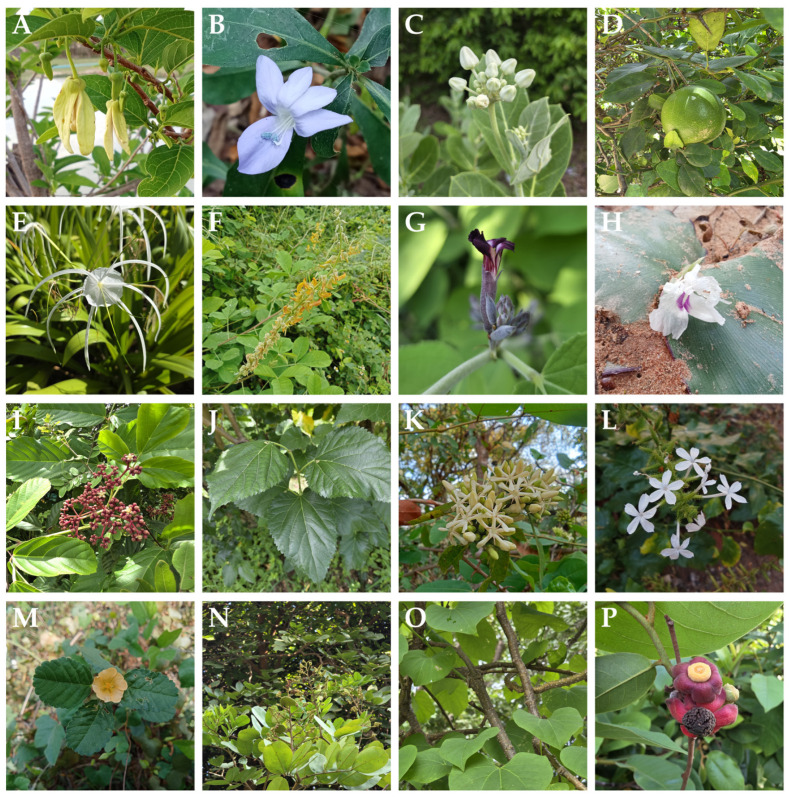
Representative antipyretic medicinal plants documented in Yasothon Province, northeastern Thailand: (**A**) *Annona squamosa* L.; (**B**) *Barleria strigosa* Willd.; (**C**) *Calotropis gigantea* (L.) W.T. Aiton; (**D**) *Citrus* × *aurantiifolia* (Christm.) Swingle; (**E**) *Crinum asiaticum* L.; (**F**) *Crotalaria pallida* Aiton; (**G**) *Helicteres hirsuta* Lour.; (**H**) *Kaempferia marginata* Carey ex Roscoe; (**I**) *Leea rubra* Blume; (**J**) *Morus alba* L.; (**K**) *Oxyceros horridus* Lour.; (**L**) *Plumbago zeylanica* L.; (**M**) *Sida rhombifolia* L.; (**N**) *Sindora siamensis* Teijsm. ex Miq.; (**O**) *Tinospora crispa*; (L.) Hook.f. & Thomson; (**P**) *Uvaria rufa* Blume. Photos by Tammanoon Jitpromma.

Patterns of generic diversity differed slightly from species diversity. Fabaceae also showed the highest generic richness with 14 genera, followed by Rubiaceae with eight genera and Annonaceae with seven genera. Rutaceae and Malvaceae each comprised six genera, whereas Euphorbiaceae and Phyllanthaceae were represented by five genera each. Apocynaceae and Asteraceae contained five and four genera, respectively, while Vitaceae included three genera and Zingiberaceae three genera. In contrast, although Ebenaceae was represented by five species, all belonged to a single genus, indicating relatively low generic diversity within the family. Representative antipyretic medicinal plants are shown in Figure 2.

### 2.2. Composition of Antipyretic Medicinal Plants

**Figure 3:**
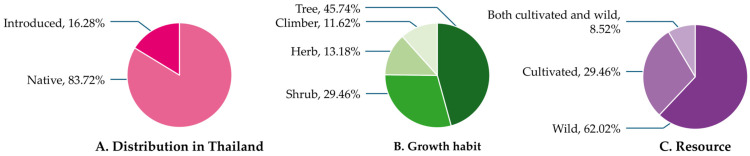
Composition of antipyretic medicinal plants documented in Yasothon Province, northeastern Thailand, showing (**A**) distribution in Thailand, (**B**) growth habit, and (**C**) resource types.

Of the 129 antipyretic medicinal plant species documented, 108 (83.72%) were native to Thailand, whereas 21 (16.28%) were introduced (Figure 3).

For example, *Aegle marmelos* is native to South and Southeast Asia, including India, Nepal, Pakistan, Bangladesh, and the Western Himalayas. *Cymbopogon citratus* originates from tropical Asia, particularly India and Sri Lanka. Similarly, *Curcuma zedoaria* is widely distributed in South and Southeast Asia, including India, Myanmar, China (South-Central region), Nepal, Sri Lanka, Vietnam, and the Eastern Himalayas. These species are now widely cultivated and naturalized in many tropical regions, including Thailand, due to their high medicinal and economic value.

The recorded flora comprised four major growth forms. Trees were the most abundant, with 59 species (45.74%), followed by shrubs (38 species, 29.46%). Herbs and climbers were less frequently represented, accounting for 17 (13.18%) and 15 species (11.62%), respectively (Figure 3).

Based on resource type, wild species predominated with 80 species (62.02%), while 38 species (29.46%) were cultivated. An additional 11 species (8.52%) occurred in both wild and cultivated habitats (Figure 3).

### 2.3. Used Parts of Antipyretic Medicinal Plants

**Figure 4:**
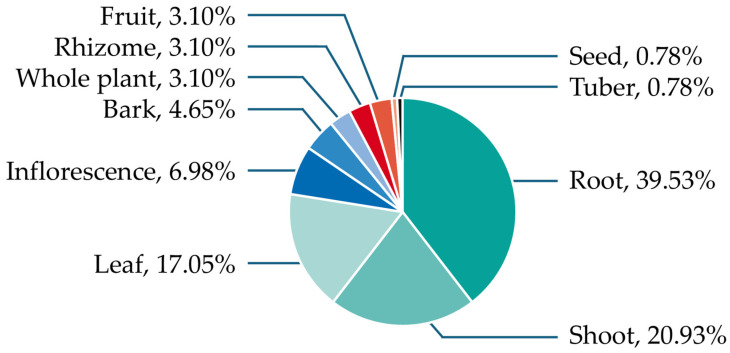
Plant parts used in traditional antipyretic remedies documented in Yasothon Province, northeastern Thailand.

Roots were the most frequently used plant part in the preparation of traditional antipyretic remedies, represented by 51 species (39.53%), followed by shoots (27 species, 20.93%) and leaves (22 species, 17.05%) (Figure 4). Inflorescences accounted for 9 species (6.98%), while bark was used in 6 species (4.65%). Fruits, rhizomes, and whole plants were each represented by 4 species (3.10%), whereas seeds and tubers were the least utilized plant parts, with one species each (0.78%).

### 2.4. Condition of Plant Material of Antipyretic Medicinal Plants

**Figure 5:**
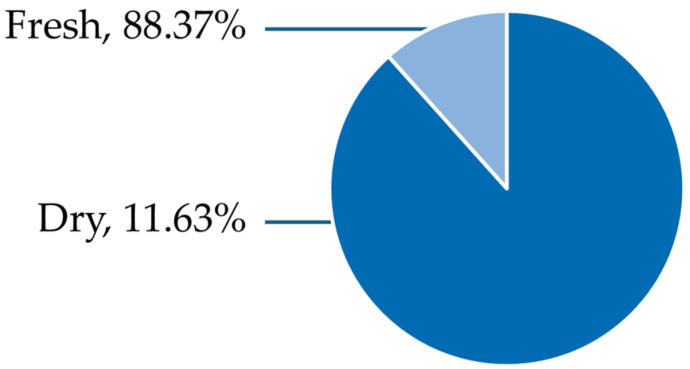
Condition of plant materials used in traditional antipyretic remedies documented in Yasothon Province, northeastern Thailand.

Fresh plant materials were predominantly used in the preparation of traditional antipyretic remedies, accounting for 114 species (88.37%), whereas only 15 species (11.63%) were used in dried form (Figure 5).

### 2.5. Preparation Methods of Antipyretic Medicinal Plants

**Figure 6:**
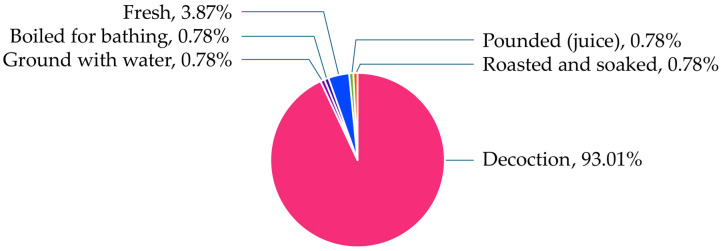
Preparation methods of antipyretic medicinal plants documented in Yasothon Province, northeastern Thailand.

Decoction was the predominant method used for preparing antipyretic remedies, accounting for 120 species (93.01%) (Figure 6). Fresh consumption was recorded for five species (3.87%), whereas boiling for bathing, grinding with water, pounding to extract juice, and roasting followed by soaking were each documented for one species (0.78%).

### 2.6. Fidelity Level (%FL) of Antipyretic Medicinal Plants

The fidelity level (FL) of the documented antipyretic medicinal plants ranged from 5.000% to 100.000% (Table 1 and Supplementary Table S1). A total of 25 species exhibited an FL value of 100.000%, indicating that all informants who cited these plants associated them specifically with fever treatment. Among these, *Symplocos racemosa* had the highest number of use citations (I_u_ = 9), followed by *Alpinia zerumbet*, *Mallotus philippensis*, and *Myxopyrum smilacifolium* subsp. *confertum* (I_u_ = 8 each). *Gnetum montanum* and *Pterospermum lanceifolium* were each supported by seven informants, whereas *Antidesma sootepense*, *Combretum pilosum*, *Diospyros ferrea*, *Gnetum macrostachyum*, and *Hymenodictyon orixense* were each cited by six informants. The remaining species with FL values of 100.00% were represented by two to five informants (Supplementary Table S1).

Among the species with FL values below 100.000%, 29 species showed high fidelity levels (50.000–99.999%). Representative examples included *Cissus hastata* (72.727%), *Ampelocissus martini* (71.429%), *Barleria strigosa* (70.000%), *Diospyros ehretioides* and *Pandanus odorifer* (66.667% each), and *Harrisonia perforata* (63.636%).

A further 53 species exhibited moderate fidelity levels (25.000–49.999%), including *Dillenia ovata* and *Polyalthia debilis* (46.154% each); *Cinnamomum verum*, *Cyanthillium cinereum, Embelia ribes*, *Mansonia gagei*, and *Terminalia chebula* (42.857% each); and *Diospyros decandra* and *Uvaria rufa* (41.667% each).

In contrast, 22 species were classified as having low fidelity levels (<25.00%). These included *Carthamus tinctorius* and *Goniothalamus laoticus* (23.529% each); *Alyxia schlechteri* and *Annona squamosa* (22.222% each); *Afzelia xylocarpa* and *Senna occidentalis* (20.000% each); and *Phyllanthus emblica*, which showed the lowest FL value (5.000%).

### 2.7. Conservation Status of Recorded Antipyretic Medicinal Plants

**Figure 7:**
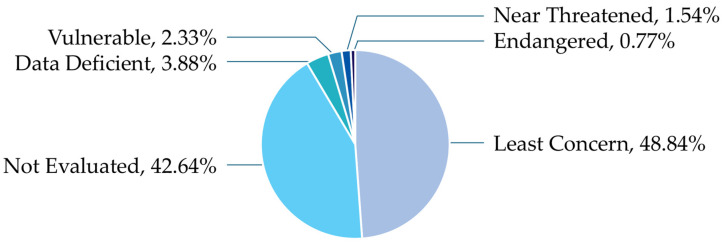
Percentage distribution of antipyretic medicinal plant species across IUCN conservation status categories recorded in Yasothon Province, northeastern Thailand.

Conservation assessment based on the IUCN Red List showed that nearly half of the recorded antipyretic medicinal plants were classified as Least Concern (LC), comprising 63 species (48.84%), whereas 55 species (42.64%) had not yet been evaluated (NE). A smaller proportion of species were categorized as Data Deficient (DD) (5 species, 3.88%), Vulnerable (VU) (3 species, 2.33%), and Near Threatened (NT) (2 species, 1.54%). Only one species (0.77%) was classified as Endangered (EN) (Figure 7).

## 3. Discussion

### 3.1. Diversity and Traditional Knowledge of Antipyretic Medicinal Plants

Fever is one of the most common symptoms encountered in rural communities and has long been managed using plant-based remedies before the widespread availability of modern healthcare services [22]. The extensive diversity of antipyretic medicinal plants documented in the present study reflects a well-developed traditional healthcare system in which local people possess multiple therapeutic options for managing the same ailment [23]. Rather than relying on a single medicinal species, communities maintain a broad repertoire of herbal remedies that can be selected according to seasonal availability, accessibility, and accumulated empirical experience [24,25,26]. Such diversity may also enhance the resilience of traditional healthcare systems by allowing households to adapt treatment practices when particular species are seasonally unavailable, locally scarce, or difficult to access.

Previous ethnobotanical studies conducted in Yasothon Province have documented useful plants and medicinal knowledge in selected localities, but these investigations were generally focused on edible plants or broader categories of traditional plant use rather than specifically on antipyretic remedies [27,28]. Moreover, earlier studies were geographically restricted to certain communities and did not survey the province as a whole. The markedly larger number of antipyretic species recorded in the present study should therefore be interpreted in relation to differences in research scope, thematic focus, and geographic coverage rather than as a direct discrepancy with previous reports. By concentrating specifically on fever-related medicinal plants and drawing evidence from communities across nine districts of Yasothon Province, the present study was able to document a much wider range of species and associated ethnomedicinal knowledge. This finding highlights the value of ailment-specific ethnobotanical investigations as a complementary approach to broader surveys, because focused studies can reveal specialized domains of traditional medicinal knowledge that may remain underrepresented when medicinal plant use is recorded only as part of general ethnobotanical inventories [3].

The predominance of Fabaceae, Annonaceae, Rutaceae, Rubiaceae, and Malvaceae further demonstrates the important contribution of species-rich families to local healthcare systems. Species within these families are widely reported to contain diverse classes of bioactive secondary metabolites, including flavonoids, alkaloids, terpenoids, phenolic compounds, and saponins, which are associated with antipyretic, anti-inflammatory, antioxidant, and antimicrobial activities. In the present study, several documented medicinal species from these families are traditionally used for fever treatment, supporting their ethnomedicinal relevance in local healthcare practices [29,30,31,32,33]. The repeated selection of plants from these families suggests that medicinal plant use is influenced not only by species availability but also by long-term cultural experience and the continued transmission of traditional ecological knowledge across generations [34,35]. At the same time, the concentration of antipyretic remedies within a limited number of taxonomic families may indicate that plant selection is not random but shaped by repeated observation of therapeutic effectiveness within culturally familiar plant groups. In this sense, the antipyretic flora of Yasothon Province reflects not only the botanical diversity of the region but also a culturally filtered body of medicinal knowledge refined through long-term interaction between local communities and their surrounding environments.

### 3.2. Plant Selection and Preparation Strategies

The predominance of woody species and wild plant resources highlights the intimate relationship between traditional healthcare practices and the surrounding ecosystems of Yasothon Province [36]. Community forests, dry dipterocarp forests, secondary vegetation, and rice-field margins function not only as reservoirs of biodiversity but also as living pharmacies that continually supply medicinal resources for rural communities [37]. The reliance on naturally occurring species suggests that the selection of antipyretic plants is strongly influenced by long-term interactions between local people and their environments, where ecological availability, accumulated experience, and cultural transmission collectively shape medicinal plant use [38].

Roots constituted the most frequently utilized plant part, reflecting a widespread traditional belief that underground organs possess concentrated therapeutic properties capable of alleviating internal disorders such as fever [39]. This preference is unlikely to be arbitrary but instead represents the outcome of a long process of empirical selection in which medicinal efficacy has been validated through repeated observation and intergenerational knowledge transfer [40]. From an ecological perspective, however, the extensive use of roots presents a potential conservation concern because root harvesting is inherently destructive and can reduce individual survival, population recruitment, and long-term species persistence, particularly for slow-growing wild taxa. Integrating traditional knowledge with sustainable harvesting practices and community-based cultivation programs may therefore be essential for maintaining both medicinal plant resources and the cultural practices associated with their use [41,42].

Decoction was overwhelmingly the principal method of preparation, indicating a remarkable consistency in traditional medicinal practice despite the taxonomic diversity of the recorded species. Rather than representing a simple preparation technique, decoction reflects a culturally established extraction strategy that is practical, inexpensive, and readily applicable under household conditions [43,44]. Boiling facilitates the release of water-soluble bioactive constituents while simultaneously reducing microbial contamination and potentially detoxifying certain plant materials, thereby enhancing both safety and therapeutic reliability [45,46]. The predominance of fresh plant materials further highlights the close integration of traditional medicine with local dietary practices. Several antipyretic species are eaten raw as fresh vegetables, reflecting a food–medicine continuum in which edible plants simultaneously provide nutritional benefits and contribute to fever management [47]. Such dual-purpose utilization demonstrates how traditional healthcare is embedded in everyday life, facilitating the continuous transmission of ethnomedicinal knowledge while promoting the sustainable use of locally available plant resources [48].

### 3.3. Fidelity Level and Informant Agreement in Antipyretic Plant Use

Fidelity level (FL) analysis provides insight into the specificity of traditional medicinal knowledge by indicating the extent to which a plant species is associated with fever treatment among the informants who reported it. In the present study, 25 species showed an FL value of 100%, meaning that all informants who cited these species referred to them specifically for the treatment of fever. This pattern suggests that, within the local ethnomedicinal system of Yasothon Province, these species are strongly linked to antipyretic use rather than being broadly distributed across multiple therapeutic categories [49,50].

Species showing both complete fidelity and relatively high numbers of informants, such as *Symplocos racemosa*, *Alpinia zerumbet*, *Mallotus philippensis*, and *Myxopyrum smilacifolium* subsp. *confertum*, were consistently reported for fever treatment by multiple informants. Their relatively high citation frequencies, together with complete fidelity, indicate a high level of agreement regarding their antipyretic use among the informants who cited these species. From an ethnopharmacological perspective, species that combine complete fidelity with relatively high citation frequencies may represent useful candidates for future phytochemical and pharmacological investigation [51,52,53,54].

However, FL values should be interpreted with caution, particularly when complete fidelity is based on only a small number of informants. In such cases, FL = 100% indicates agreement only among the individuals who cited that species and does not necessarily imply broad cultural importance at the community level. Species reported by only a few informants may instead represent localized, household-based, or specialist knowledge retained by particular healers or experienced practitioners [54]. Their documentation nevertheless remains important, as such knowledge forms part of the biocultural heritage of the province and may otherwise be lost through ongoing social and cultural change [55].

Complete fidelity also does not imply that a species is used exclusively for fever in other cultural or geographic settings. Rather, FL reflects the therapeutic role assigned to a plant within a specific local medical system. For example, *Alpinia zerumbet* showed an FL value of 100% in the present study, indicating that all informants who cited the species associated it with fever treatment. In contrast, previous ethnobotanical research in Kalasin Province recorded its use for gastrointestinal disorders, infections, and musculoskeletal conditions [56], while broader reviews have documented additional uses such as antihypertensive, anti-inflammatory, expectorant, digestive, and rheumatism-related applications [57]. These differences highlight the context-dependent nature of medicinal plant knowledge, in which the same species may assume different therapeutic roles according to local health needs, cultural experience, and patterns of knowledge transmission [58].

### 3.4. Conservation and Future Perspectives

The conservation assessment of antipyretic medicinal plants recorded in Yasothon Province showed that nearly half of the documented species were classified as Least Concern (LC), while a substantial proportion had not yet been evaluated (NE) under the IUCN Red List framework. Smaller numbers of species were assigned to Data Deficient (DD), Vulnerable (VU), Near Threatened (NT), and Endangered (EN) categories. These findings indicate that, although many antipyretic medicinal plants currently do not appear to face immediate global conservation risk, a considerable number remain insufficiently assessed, limiting a more complete understanding of their long-term conservation security.

This issue becomes particularly important when considered alongside the patterns of plant use documented in the present study. Traditional antipyretic medicine in Yasothon Province remains strongly dependent on native and wild plant resources, many of which are collected directly from natural habitats. In addition, roots constituted the most frequently used plant part. Because root harvesting can damage or kill the entire plant, repeated extraction from wild populations may reduce regeneration capacity and increase local depletion pressure, especially for species that are already categorized as threatened or remain poorly evaluated [59]. The potential conservation risk is therefore shaped not only by formal threat status but also by harvesting intensity, the plant parts collected, and the degree to which local healthcare continues to rely on naturally occurring species.

Beyond its relevance to biodiversity conservation, the documentation of antipyretic medicinal plants also contributes to the safeguarding of biocultural heritage. Knowledge of fever remedies in Yasothon Province reflects long-term interactions between local communities and their surrounding environments and forms part of a broader indigenous healthcare system maintained through oral transmission and everyday practice. As modernization, land-use change, and changing healthcare behaviors continue to affect rural communities, this knowledge may become increasingly vulnerable to erosion [60].

The present inventory therefore provides an important baseline for future research and conservation planning. Species with high fidelity values and strong community recognition may serve as promising candidates for phytochemical and pharmacological investigation, while taxa that are wild-harvested, root-collected, or associated with threatened and poorly evaluated conservation categories warrant closer ecological monitoring and sustainable management. In this way, the present study not only expands ethnomedicinal knowledge of antipyretic plants in northeastern Thailand but also highlights the need to integrate traditional knowledge documentation with conservation assessment and long-term resource stewardship.

### 3.5. Study Limitations

This study has several limitations that should be considered when interpreting the findings. First, the survey was conducted exclusively in Yasothon Province, northeastern Thailand, and the documented antipyretic uses therefore reflect local ethnomedicinal knowledge within this particular cultural and ecological context. Although the study provides the first comprehensive account of antipyretic medicinal plants in the province, the findings should not be generalized to other regions of Thailand without further comparative investigation. Second, the fidelity level (FL) used in this study measures the degree of informant consensus regarding the use of individual plant species for fever treatment, but it does not demonstrate pharmacological efficacy or clinical effectiveness. High FL values indicate strong agreement within the local knowledge system rather than direct evidence of antipyretic activity. Third, the medicinal applications reported here are based on traditional knowledge transmitted through community practice and oral tradition. While such information provides an important foundation for identifying culturally significant medicinal plants, further phytochemical, pharmacological, toxicological, and clinical studies are needed to validate their bioactive properties, safety, and therapeutic potential.

## 4. Materials and Methods

### 4.1. Study Area

**Figure 8:**
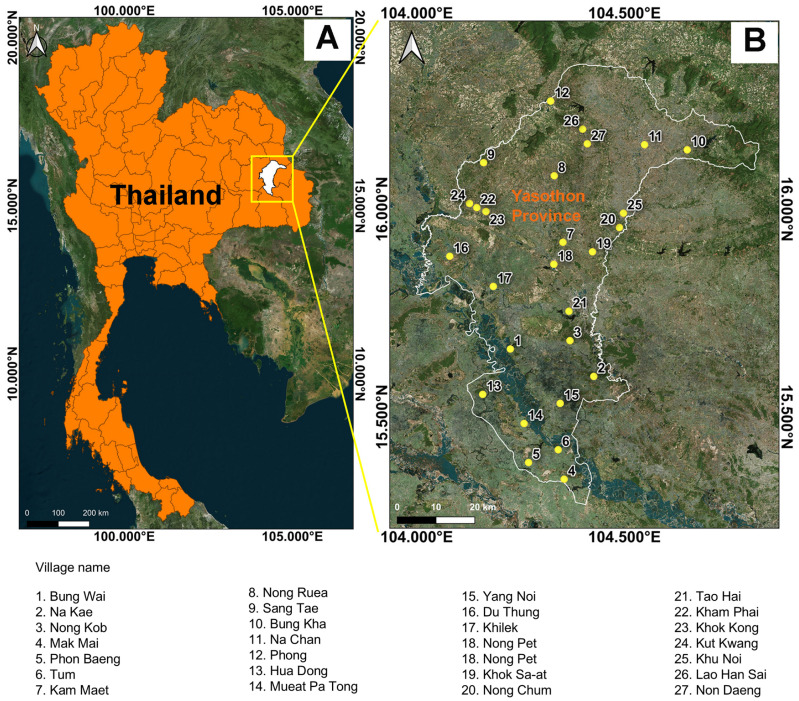
Location of the study area in Yasothon Province, northeastern Thailand: (**A**) geographical location of Yasothon Province within Thailand; (**B**) surveyed villages and informant interview sites in Yasothon Province. The map was generated using QGIS version 3.34 [62] with the WGS 84 geographic coordinate system (EPSG: 4326).

The study was conducted in Yasothon Province, located in northeastern Thailand (Isan region) (Figure 8). The province covers approximately 4161 km^2^ and lies between 15°15′–16°05′ N latitude and 104°05′–104°45′ E longitude on the Khorat Plateau, a major physiographic region characterized by lowland plains and gently undulating terrain. Yasothon Province is bordered by Mukdahan Province to the north, Amnat Charoen Province to the east, Sisaket and Ubon Ratchathani provinces to the south, and Roi Et Province to the west [61].

The northern part of the province consists mainly of plains with low hills, whereas the southern region is dominated by lowlands associated with the Chi River Basin, including ponds, wetlands, and seasonally flooded areas. These landscapes support a mosaic of ecosystems comprising rain-fed rice fields, dry dipterocarp forests, mixed deciduous forests, community forests, fallow lands, wetlands, and secondary vegetation. The province retains approximately 358 km^2^ of forest area, representing about 8.7% of its total area [63].

Yasothon Province experiences a tropical savanna climate (Köppen Aw) characterized by distinct rainy and dry seasons. The rainy season extends from May to October under the influence of the southwest monsoon, whereas the dry season occurs from November to April. Annual rainfall averages approximately 1200–1600 mm, and mean annual temperatures range from 26 to 28 °C, with maximum temperatures frequently exceeding 38 °C during the hot season (March–May). These environmental conditions strongly influence vegetation dynamics, plant phenology, and the seasonal availability of medicinal plant resources collected from forests, wetlands, agricultural landscapes, and home gardens [64,65].

The soils of Yasothon Province are predominantly sandy loam and ferralsol-derived soils associated with the Yasothon and Roi Et soil series. These soils generally exhibit low fertility, low organic matter content, and limited water-holding capacity, contributing to periodic drought stress in upland areas and temporary flooding in lowland ecosystems during the rainy season. Such environmental heterogeneity supports a wide variety of native and cultivated plant species that are traditionally utilized for medicinal purposes [66].

Yasothon Province has a predominantly rural population of approximately 522,000 inhabitants, most of whom belong to Lao-Isan cultural groups and rely primarily on agriculture, particularly rice cultivation [61,67]. Traditional medicinal knowledge remains closely integrated with local healthcare practices, where wild and cultivated plants are commonly used for the prevention and treatment of various ailments, including fever, gastrointestinal disorders, musculoskeletal diseases, skin conditions, and postpartum care. Knowledge of medicinal plant identification, harvesting, preparation, and administration is primarily transmitted orally through traditional healers, elderly community members, and family networks. However, rapid socioeconomic development, urbanization, and changing lifestyles have increasingly reduced the intergenerational transmission of this knowledge, placing valuable ethnomedicinal traditions at risk of disappearance. Consequently, Yasothon Province represents an important area for documenting traditional medicinal knowledge and preserving the biocultural heritage associated with antipyretic medicinal plants [68,69].

### 4.2. Preliminary Ethnomedicinal Survey and Key Informant Interviews

An ethnomedicinal survey was conducted in Yasothon Province between April 2024 and March 2026 to document medicinal plants used by local communities for the treatment of various ailments. For the purposes of the present study, only plant species specifically reported as antipyretic remedies were extracted from the broader medicinal dataset and analyzed in detail.

Key informants were selected using purposive sampling and included traditional healers, village elders, herbal practitioners, and community members recognized for their knowledge of medicinal plants. Semi-structured interviews were conducted to record local plant names, plant parts used, preparation methods, routes of administration, and therapeutic applications. Information obtained during the interviews was verified through repeated discussions and cross-checking among informants.

Guided field walks with key informants were carried out in natural habitats, agricultural landscapes, and home gardens to locate medicinal plant species and observe traditional harvesting practices. These field visits also provided contextual information on plant occurrence, collection sites, and local use and supported subsequent specimen collection and taxonomic verification.

The survey generated a broader inventory of medicinal plants and their therapeutic uses in Yasothon Province. From this dataset, species reported for fever treatment were selected for the present paper in order to provide a focused analysis of traditional antipyretic medicinal plants.

### 4.3. Plant Collection and Identification

Plant specimens were collected during guided field walks with key informants in community forests, dry dipterocarp forests, mixed deciduous forests, wetlands, rice-field margins, agricultural areas, and home gardens. Fertile specimens bearing flowers or fruits were collected whenever available to facilitate taxonomic identification.

Each specimen was photographed in the field and prepared following standard herbarium procedures. Voucher specimens were dried, mounted, labeled, and deposited at the Vascular Plant Herbarium, Mahasarakham University (VMSU), Kantharawichai District, Maha Sarakham Province, Thailand, for long-term preservation and future reference.

Species identification was carried out using the Flora of Thailand together with relevant taxonomic literature and botanical monographs. Scientific names, author citations, and family classifications were verified and updated using Plants of the World Online (POWO) (https://powo.science.kew.org) [70] (accessed on 15 February 2026) to ensure consistency with current taxonomic standards.

### 4.4. Conservation Status

The global conservation status of the recorded species was verified using the IUCN Red List of Threatened Species (https://www.iucnredlist.org) [71] (accessed on 5 July 2026). Scientific names standardized according to the taxonomic references used in this study were matched with their corresponding IUCN Red List categories. The IUCN Red List was used solely to indicate global conservation status and not to assess local conservation status or harvesting pressure.

### 4.5. Ethnobotanical Data Collection

A total of 108 informants from 27 villages across nine districts of Yasothon Province participated in the study (Table 2). To ensure comparable village-level representation, four knowledgeable informants were recruited from each village. The study was designed to include two male and two female informants from each village in order to capture potential gender-related variation in ethnomedicinal knowledge while maintaining balanced representation across the study area. Informants ranged in age from 20 to 68 years. All ethnobotanical interviews were conducted during February 2026.

Informants were selected using purposive sampling, with snowball sampling subsequently employed to identify additional knowledgeable participants through recommendations from the initial informants. Participant selection focused on individuals recognized within their communities as possessing practical knowledge and experience in the use of medicinal plants. Recommendations were obtained from traditional healers, Buddhist monks recognized for their knowledge of medicinal plants, village leaders, and other knowledgeable community members. The final group of participants included traditional healers, village elders, herbal practitioners, experienced plant collectors, and local residents who regularly prepared or used herbal remedies.

Semi-structured interviews, participant observation, and guided field walks were used to document ethnomedicinal information related to antipyretic plant use. Interviews were conducted in either Thai or the Lao-Isan dialect according to informant preference. The information recorded included local plant names, plant parts used, preparation methods, routes of administration, dosage forms, therapeutic applications related to fever treatment, combinations with other medicinal species, collection habitats, and seasonal availability.

Before each interview, the objectives of the study were explained to all participants, and prior informed consent was obtained. The research followed the Code of Ethics of the International Society of Ethnobiology (ISE) [72] and complied with the principles of the Nagoya Protocol on Access and Benefit-Sharing [73]. Ethical approval for the interview component of this study was granted by the Ethics Committee for Human Research, Mahasarakham University (Approval No. 045-905/2026; 21 January 2026), and all interviews were conducted after ethical approval had been obtained. Participation was voluntary, and informants were free to withdraw from the study at any stage. All information was recorded with respect for local knowledge systems and community intellectual rights.

### 4.6. Data Analysis

The present study represents a focused analysis of antipyretic medicinal plants extracted from a broader ethnomedicinal dataset documented in Yasothon Province. From the complete medicinal plant inventory recorded during fieldwork, only species reported for fever treatment were selected for detailed analysis in this paper. To evaluate the degree of informant agreement regarding the use of these species as antipyretic remedies, fidelity level (FL) was applied.

FL was calculated according to the following equation [74]:

(1) $$\mathrm{FL}\;=\;\frac{I_{p}}{I_{u}}\;\times \;100$$

where I_p_ is the number of informants who independently cited a plant species specifically for fever treatment, and I_u_ is the total number of informants who mentioned that species for any medicinal use recorded in the Yasothon ethnomedicinal survey. In this study, FL was used to assess the extent to which a medicinal plant was specifically associated with antipyretic use among the informants who reported that species. Higher FL values indicate that a greater proportion of medicinal citations for a given species were related to fever treatment, whereas lower values suggest that the species was more commonly associated with a broader range of medicinal applications beyond fever.

Because this paper focuses exclusively on one therapeutic category, FL was considered the most appropriate index for examining use specificity and informant agreement related to antipyretic plant use. However, FL does not by itself represent the overall cultural importance of a species and may be influenced by the number of informants citing that species. Therefore, FL values were interpreted together with the number of informants reporting each plant, particularly for species with complete fidelity but relatively few citations.

## 5. Conclusions

This study provides the first comprehensive documentation of traditional antipyretic medicinal plants used in Yasothon Province, northeastern Thailand, revealing a rich body of ethnomedicinal knowledge that remains actively maintained within local communities. The documented diversity of medicinal species, together with information on plant parts used, preparation methods, and fidelity levels, demonstrates the complexity and cultural significance of traditional fever management practices.

The predominance of native and wild medicinal plants highlights the close relationship between local healthcare systems and regional biodiversity, while the frequent use of roots emphasizes the importance of promoting sustainable harvesting and conservation strategies. The widespread reliance on decoction and the use of fresh edible plants further illustrate how medicinal practices are deeply integrated into everyday life and local food traditions.

Species exhibiting high fidelity levels represent culturally important antipyretic remedies with strong community consensus and should be considered priority candidates for future phytochemical, pharmacological, and toxicological investigations. At the same time, species showing complete fidelity but supported by relatively few informants preserve unique and localized ethnomedicinal knowledge that warrants further documentation before it is lost through ongoing social and cultural change.

Overall, this study expands the ethnobotanical knowledge of Yasothon Province and provides a valuable baseline for biodiversity conservation, the preservation of traditional medicinal knowledge, and the scientific validation of plant-based antipyretic remedies. The findings also contribute to a broader understanding of traditional fever management in Thailand and may support the sustainable utilization and future development of medicinal plant resources.

## Figures and Tables

**Table 1:** Ethnobotanical characteristics and fidelity level (FL) of antipyretic medicinal plants documented in Yasothon Province, northeastern Thailand.

Family	Scientific Name	Vernacular Name	DiT	HB	RS	Used Parts	CoP	Preparation	I_p_	I_u_	FL	IUCN	VN
Acanthaceae	*Barleria strigosa* Willd.	สังกรณี (Sangkarani)	Nt	Hb	Wd	RT	F	Prepared as a decoction, then filtered, and consumed orally (Decoction).	7	10	70.000	NE	TJY525
Amaryllidaceae	*Crinum asiaticum* L.	พลับพลึง (Phlapphlueng)	Nt	Hb	Ct	LV	F	Decoction	2	12	16.667	NE	TJY306
Anacardiaceae	*Mangifera indica* L.	มะม่วง (Mamuang)	Nt	Tr	Ct	BK	F	Decoction	6	21	28.571	DD	TJY0103
Annonaceae	*Annona squamosa* L.	น้อยหน่า (Noi Na)	It	Tr	Ct	LV	F	Decoction	4	18	22.222	LC	TJY216
Annonaceae	*Cananga brandisiana* (Pierre) Saff.	สะแกแสง (Sakae Saeng)	Nt	Tr	Wd	WP	F	Pounded, and the juice is extracted and consumed orally.	3	12	25.000	LC	TJ-F0012
Annonaceae	*Goniothalamus laoticus* (Finet & Gagnep.) Bân	ข้าวหลามดง (Khao Lam Dong)	Nt	Tr	Wd	SM	F	Decoction	4	17	23.529	NE	TJ-F0030
Annonaceae	*Mitrephora maingayi* Hook.f. & Thomson	นางแดง (Nang Daeng)	Nt	Tr	Wd	RT	F	Decoction	3	3	100.000	NE	TJY541
Annonaceae	*Polyalthia debilis* (Pierre) Finet & Gagnep.	ก้นครก (Kon Khrok)	Nt	Sh	Wd	RT	D	Decoction	6	13	46.154	NE	TJ-F0050
Annonaceae	*Uvaria hirsuta* Jack	เงาะป่า (Ngo Pa)	Nt	Cl	Wd	SM	F	Decoction	3	18	16.667	NE	TJY539
Annonaceae	*Uvaria rufa* Blume	ผีผ่วน (Phi Phuan)	Nt	Cl	Wd	SM	D	Decoction	5	12	41.667	NE	TJ-F0059
Annonaceae	*Uvaria siamensis* (Scheff.) L.L.Zhou, Y.C.F.Su & R.M.K.Saunders	ลำดวน (Lamduan)	Nt	Sh	Bt	IF	D	Decoction	3	19	15.789	LC	TJ-F0047
Annonaceae	*Xylopia vielana* Pierre	กล้วยน้อย (Kluai Noi)	Nt	Tr	Wd	RT	D	Decoction	2	6	33.333	LC	TJ-F0004
Apocynaceae	*Alyxia schlechteri* H.Lév.	ตังตุ่น (Tang Tun)	It	Sh	Wd	IF	F	Decoction	2	9	22.222	NE	TJ-F0034
Apocynaceae	*Beaumontia murtonii* Craib	กำลังช้างสาร (Kamlang Chang San)	Nt	Cl	Wd	RT	F	Decoction	3	11	27.273	NE	TJY545
Apocynaceae	*Calotropis gigantea* (L.) W.T. Aiton	รัก (Rak)	Nt	Hb	Wd	IF	F	Decoction	6	10	60.000	NE	TJY293
Apocynaceae	*Stephanotis volubilis* (L.f.) S.Reuss, Liede & Meve	กระทงหมาบ้า (Krathong Ma Ba)	Nt	Cl	Wd	RT	F	Decoction	8	14	57.143	NE	TJY552
Apocynaceae	*Tabernaemontana bufalina* Lour.	พริกป่า (Phrik Pa)	Nt	Sh	Wd	RT	F	Decoction	4	12	33.333	LC	TJY556
Arecaceae	*Areca catechu* L.	หมาก (Mak)	It	Tr	Ct	LV	F	Boiled and used as a bathing solution.	6	10	60.000	LC	TJY286
Asparagaceae	*Dracaena angustifolia* (Medik.) Roxb.	คอนแคน (Khon Khaen)	Nt	Sh	Wd	SM	F	Decoction	5	5	100.000	NE	TJY314
Asteraceae	*Blumea balsamifera* (L.) DC.	หนาดใหญ่ (Nat Yai)	Nt	Sh	Wd	LV	F	Decoction	5	10	50.000	LC	TJY0193
Asteraceae	*Carthamus tinctorius* L.	คำฝอย (Kham Foi)	It	Hb	Ct	IF	D	Decoction	4	17	23.529	NE	TJY566
Asteraceae	*Chrysanthemum indicum* L.	เก๊กฮวย (Kek Huai)	It	Hb	Ct	IF	D	Decoction	6	16	37.500	NE	TJY621
Asteraceae	*Cyanthillium cinereum* (L.) H.Rob.	หญ้าดอกขาว (Ya Dok Khao)	Nt	Hb	Wd	SM	F	Decoction	6	14	42.857	NE	TJY633
Bixaceae	*Bixa orellana* L.	คำแสด (Kham Saet)	It	Tr	Ct	LV	F	Decoction	3	12	25.000	LC	TJY562
Capparaceae	*Capparis micracantha* DC.	ชิงชี่ (Ching Chi)	Nt	Sh	Wd	RT	F	Decoction	2	14	14.286	LC	TJY679
Capparaceae	*Crateva magna* (Lour.) DC.	กุ่มน้ำ (Kum Nam)	Nt	Tr	Ct	BK	D	Decoction	4	13	30.769	LC	TJY632
Capparaceae	*Crateva religiosa* G.Forst.	ผักกุ่ม (Phak Kum)	Nt	Tr	Ct	BK	D	Decoction	4	14	28.571	LC	TJY564
Combretaceae	*Combretum pilosum* Roxb. ex G.Don	งวงสุม (Nguang Sum)	Nt	Cl	Wd	RT	F	Decoction	6	6	100.000	NE	TJY565
Combretaceae	*Terminalia chebula* Retz.	บักส้มมอ (Bak Som Mo)	Nt	Tr	Wd	FT	F	Eaten fresh.	3	7	42.857	LC	TJ-F0036
Connaraceae	*Ellipanthus tomentosus* Kurz	ตานกกด (Ta Nok Kot)	Nt	Tr	Wd	SM	F	Decoction	2	8	25.000	LC	TJY693
Crypteroniaceae	*Crypteronia paniculata* Blume	กะอาม (Ka Am)	Nt	Tr	Wd	LV	F	Decoction	3	3	100.000	LC	TJY694
Dilleniaceae	*Dillenia hookeri* Pierre	ส้านดิน (San Din)	Nt	Tr	Wd	SM	F	Decoction	2	8	25.000	LC	TJY630
Dilleniaceae	*Dillenia ovata* Wall. ex Hook.f. & Thomson	ส้านใบเล็ก (San Bai Lek)	Nt	Tr	Wd	SM	F	Decoction	6	13	46.154	LC	TJ-F0037
Dioscoreaceae	*Tacca leontopetaloides* (L.) Kuntze	เท้ายายม่อม (Thao Yai Mom)	Nt	Hb	Wd	RT	F	Decoction	4	10	40.000	LC	TJY379
Dipterocarpaceae	*Anthoshorea roxburghii* (G.Don) P.S.Ashton & J.Heck.	กะยอม (Kayom)	Nt	Tr	Wd	SM	F	Decoction	4	4	100.000	LC	TJY648
Ebenaceae	*Diospyros decandra* Lour.	จันทร์ (Chan)	Nt	Tr	Wd	SM	F	Decoction	5	12	41.667	LC	TJ-F0045
Ebenaceae	*Diospyros ehretioides* Wall. ex G.Don	ตับเต่าใหญ่ (Tap Tao Yai)	Nt	Tr	Wd	SM	F	Ground with water and taken orally.	6	9	66.667	LC	TJY627
Ebenaceae	*Diospyros ferrea* (Willd.) Bakh.	ขี้หนู (Khi Nu)	Nt	Tr	Wd	RT	F	Decoction	6	6	100.000	LC	TJY655
Ebenaceae	*Diospyros filipendula* Pierre ex Lecomte	บักคันจ้อง (Bak Khan Chong)	Nt	Tr	Wd	RT	F	Decoction	7	13	53.846	LC	TJY451, TJ-F0054
Ebenaceae	*Diospyros undulata* Wall. ex G.Don	คำดีควาย (Kham Di Khwai)	Nt	Tr	Wd	SM	F	Decoction	4	4	100.000	LC	TJY649
Euphorbiaceae	*Acalypha spiciflora* Burm.f.	ดีหมี (Di Mi)	It	Tr	Wd	WP	F	Decoction	2	5	40.000	NE	TJY664
Euphorbiaceae	*Cladogynos orientalis* Zipp. ex Span.	เเจตพังคี (Chet Phangkhi)	Nt	Sh	Ct	RT	F	Decoction	4	4	100.000	LC	TJY656
Euphorbiaceae	*Croton crassifolius* Geiseler	พังคีน้อย (Phangkhi Noi)	Nt	Sh	Ct	RT	F	Decoction	3	9	33.333	NE	TJY653
Euphorbiaceae	*Croton persimilis* Müll.Arg.	เปล้าใหญ่ (Plao Yai)	Nt	Tr	Ct	LV	F	Decoction	1	8	12.500	LC	TJY671
Euphorbiaceae	*Mallotus philippensis* (Lam.) Müll.Arg.	จำปีป่า (Champi Pa)	Nt	Tr	Wd	RT	F	Decoction	8	8	100.000	LC	TJY643
Euphorbiaceae	*Strophioblachia fimbricalyx* Boerl.	ครุย (Khrui)	Nt	Sh	Wd	RT	F	Decoction	5	5	100.000	LC	TJY677
Fabaceae	*Afzelia xylocarpa* (Kurz) Craib	มะค่าโมง (Makha Mong)	Nt	Tr	Bt	RT	F	Decoction	4	20	20.000	EN	TJY465
Fabaceae	*Cassia fistula* L.	คูณ (Khun)	It	Tr	Ct	IF	F	Decoction	6	11	54.545	LC	TJY0052, TJY295
Fabaceae	*Clitoria ternatea* L.	อัญชัน (Anchan)	It	Cl	Bt	IF	F	Eaten fresh.	4	22	18.182	NE	TJY0053, TJY231, TJY300, TJY459
Fabaceae	*Crotalaria pallida* Aiton	หิ่งเม่น (Hing Men)	Nt	Hb	Wd	RT	F	Decoction	7	21	33.333	NE	TJY604
Fabaceae	*Dalbergia cultrata* T.S.Ralph	กระพี้เขาควาย (Kraphi Khao Khwai)	Nt	Tr	Wd	SM	F	Decoction	4	7	57.143	NT	TJY651
Fabaceae	*Flemingia stricta* Roxb.	หญ้าหางเสือ (Ya Hang Suea)	Nt	Sh	Wd	RT	F	Decoction	2	11	18.182	NE	TJY635
Fabaceae	*Imbralyx leucanthus* var. *leucanthus*	สะท้อน (Sathon)	Nt	Hb	Wd	SM	F	Decoction	5	5	100.000	NE	TJY667
Fabaceae	*Phanera strychnifolia* (Craib) K.W.Jiang, S.R.Gu & T.Y.Tu	ย่านางแดง (Yanang Daeng)	Nt	Cl	Wd	RT	F	Decoction	5	18	27.778	NE	TJY606
Fabaceae	*Phyllodium longipes* (Craib) Schindl.	เกล็ดลิ่นใหญ่ (Klet Lin Yai)	Nt	Sh	Wd	RT	F	Decoction	7	13	53.846	NE	TJY636
Fabaceae	*Phyllodium pulchellum* (L.) Desv.	เกล็ดปลาช่อน (Klet Pla Chon)	Nt	Sh	Wd	LV	F	Decoction	5	10	50.000	LC	TJY339
Fabaceae	*Senna occidentalis* (L.) Link	ชุมเห็ดเล็ก (Chum Het Lek)	It	Sh	Wd	RT	F	Decoction	2	10	20.000	LC	TJY607
Fabaceae	*Senna tora* (L.) Roxb.	ชุมเห็ดไทย (Chum Het Thai)	It	Sh	Wd	RT	F	Decoction	4	14	28.571	NE	TJY638
Fabaceae	*Sindora siamensis* Teijsm. ex Miq.	บักแต้ (Bak Tae)	Nt	Tr	Bt	RT	F	Decoction	5	9	55.556	LC	TJY417, TJ-F0069
Fabaceae	*Sophora exigua* Craib	แผ่นดินเย็น (Phaen Din Yen)	Nt	Sh	Wd	RT	F	Decoction	3	10	30.000	NE	TJY672
Fabaceae	*Tamarindus indica* L.	บักขาม (Bak Kham)	It	Tr	Ct	SE	F	The seeds are roasted, the shells removed, and the kernels soaked in salt water until soft before consumption.	4	13	30.769	LC	TJY0065, TJY352
Fabaceae	*Xylia xylocarpa* var. *kerrii* (Craib & Hutch.) I.C.Nielsen	แดง (Daeng)	Nt	Tr	Bt	BK	F	Decoction	3	11	27.273	NE	TJY590
Gnetaceae	*Gnetum macrostachyum* Hook.f.	เมื่อยแดง (Mueai Daeng)	Nt	Cl	Bt	RT	F	Decoction	6	6	100.000	LC	TJY701
Gnetaceae	*Gnetum montanum* Markgr.	เมื่อยขาว (Mueai Khao)	Nt	Cl	Bt	RT	F	Decoction	7	7	100.000	LC	TJY702
Irvingiaceae	*Irvingia malayana* Oliv. ex A.W.Benn.	บักบก (Bak Bok)	Nt	Tr	Ct	BK	F	Decoction	4	25	16.000	LC	TJY0181, TJY422, TJ-F0061
Lamiaceae	*Clerodendrum schmidtii* C.B.Clarke	ใต้ใบ (Tai Bai)	Nt	Sh	Wd	LV	F	Decoction	2	5	40.000	NE	TJY662
Lauraceae	*Cinnamomum verum* J.Presl	อบเชย (Opchoei)	It	Tr	Bt	LV	F	Decoction	6	14	42.857	VU	TJY592
Loganiaceae	*Strychnos nux-blanda* A.W.Hill	ตูมกาขาว (Tum Ka Khao)	Nt	Tr	Wd	LV	F	Decoction	2	16	12.500	LC	TJY478
Malvaceae	*Helicteres hirsuta* Lour.	ปอเต่าไห้ (Po Tao Hai)	Nt	Sh	Wd	SM	F	Decoction	5	14	35.714	NE	TJY707
Malvaceae	*Hibiscus sabdariffa* L.	กระเจี๊ยบแดง (Krachiap Daeng)	It	Hb	Ct	FT	D	Decoction	5	18	27.778	NE	TJY587
Malvaceae	*Mansonia gagei* J.R.Drumm.	จันทน์ชะมด (Chan Chamot)	Nt	Tr	Ct	SM	F	Decoction	3	7	42.857	NE	TJY0186
Malvaceae	*Pterospermum lanceifolium* Roxb. ex DC.	ตากวาง (Ta Kwang)	Nt	Tr	Ct	RT	F	Decoction	7	7	100.000	LC	TJY600
Malvaceae	*Sida rhombifolia* L.	หญ้าขัด (Ya Khat)	Nt	Sh	Wd	LV	F	Decoction	6	17	35.294	NE	TJY706
Malvaceae	*Sterculia guttata* Roxb.	ปอแดง (Po Daeng)	Nt	Tr	Wd	RT	F	Decoction	2	2	100.000	LC	TJY698
Malvaceae	*Sterculia lanceolata* Cav.	ลิ้นเสือ (Lin Suea)	Nt	Sh	Wd	RT	F	Decoction	4	4	100.000	LC	TJY712
Melastomataceae	*Memecylon edule* Roxb.	บักพลองเหมือด (Bak Phlong Mueat)	Nt	Tr	Wd	SM	F	Decoction	3	13	23.077	LC	TJ-F0052
Melastomataceae	*Memecylon grande* Retz.	เหมือด (Mueat)	It	Tr	Wd	RT	F	Decoction	3	3	100.000	VU	TJY709
Meliaceae	*Azadirachta indica* A.Juss.	สะเดา (Sadao)	Nt	Tr	Ct	IF	F	Eaten fresh.	3	11	27.273	LC	TJY0125, TJY218
Menispermaceae	*Tiliacora triandra* (Colebr.) Diels	ย่านาง (Yanang)	Nt	Cl	Ct	RT	F	Decoction	6	12	50.000	NE	TJY0144, TJY377, TJY273
Menispermaceae	*Tinospora crispa* (L.) Hook.f. & Thomson	บอระเพ็ด (Boraphet)	Nt	Cl	Wd	SM	F	Decoction	7	17	41.176	NE	TJY680
Moraceae	*Ficus hispida* L.f.	บักเดื่อปล้อง (Bak Duea Plong)	Nt	Tr	Wd	FT	F	Eaten fresh.	6	16	37.500	LC	TJ-F0046
Moraceae	*Ficus racemosa* L.	บักเดื่อชุมพร (Bak Duea Chumphon)	Nt	Tr	Wd	RT	D	Decoction	6	12	50.000	LC	TJY0106, TJY425, TJ-F0029
Moraceae	*Morus alba* L.	หม่อน (Mon)	It	Sh	Ct	LV	F	Decoction	5	10	50.000	LC	TJY0107
Oleaceae	*Myxopyrum smilacifolium* subsp. *confertum* (Kerr) P.S.Green	เครือเขาเลี่ยม (Khruea Khao Liam)	Nt	Cl	Wd	RT	F	Decoction	8	8	100.000	NE	TJY681
Orchidaceae	*Eulophia attenuata* (Griff.) M.W.Chase, Kumar & Schuit.	ว่านจูงนาง (Wan Chung Nang)	Nt	Hb	Wd	TU	F	Decoction	5	8	62.500	NE	TJY508
Pandanaceae	*Pandanus odorifer* (Forssk.) Kuntze	เตยทะเล (Toei Thale)	Nt	Tr	Ct	RT	F	Decoction	6	9	66.667	LC	TJY613
Phyllanthaceae	*Antidesma sootepense* Craib	บักเม่าสาย (Bak Mao Sai)	Nt	Tr	Ct	RT	F	Decoction	6	6	100.000	LC	TJY506
Phyllanthaceae	*Aporosa villosa* (Lindl.) Baill.	เหมือดโลด (Mueat Lot)	Nt	Tr	Wd	SM	F	Decoction	5	10	50.000	LC	TJY596
Phyllanthaceae	*Breynia glauca* Craib	จ้าสีเสียด (Cha Sisiat)	Nt	Tr	Wd	RT	F	Decoction	4	10	40.000	NE	TJY682
Phyllanthaceae	*Bridelia harmandii* Gagnep.	ซำซาเตี้ย (Samsa Tia)	Nt	Sh	Wd	RT	F	Decoction	6	11	54.545	NE	TJY710
Phyllanthaceae	*Phyllanthus collinsiae* Craib	ค่างเต้น (Khang Ten)	Nt	Sh	Wd	RT	F	Decoction	4	13	30.769	DD	TJY486
Phyllanthaceae	*Phyllanthus emblica* L.	บักขามป้อม (Bak Kham Pom)	Nt	Tr	Bt	RT	F	Decoction	1	20	5.000	LC	TJY0102, TJY397, TJ-F0057
Plumbaginaceae	*Plumbago zeylanica* L.	เจตมูลเพลิงขาว (Chetmun Phloeng Khao)	Nt	Sh	Ct	RT	F	Decoction	3	3	100.000	NE	TJY532
Poaceae	*Cymbopogon citratus* (DC.) Stapf	ตะไคร้ (Takhrai)	It	Hb	Ct	LV	F	Decoction	6	11	54.545	NE	TJY0114, TJY241
Primulaceae	*Ardisia helferiana* Kurz	ส้มกุ้งขน (Som Kung Khon)	Nt	Tr	Wd	SM	F	Decoction	4	7	57.143	NE	TJY533
Primulaceae	*Embelia ribes* Burm.f.	ส้มกุ้ง (Som Kung)	Nt	Sh	Wd	RT	F	Decoction	3	7	42.857	NE	TJY597
Rubiaceae	*Canthium berberidifolium* E.T.Geddes	บักเงี่ยงดุก (Bak Ngiang Duk)	Nt	Sh	Wd	SM	F	Decoction	5	18	27.778	NE	TJY456, TJ-F0049
Rubiaceae	*Catunaregam tomentosa* (Blume ex DC.) Tirveng.	หนามแท่ง (Nam Thaeng)	Nt	Tr	Wd	LV	F	Decoction	8	14	57.143	NE	TJY512
Rubiaceae	*Dioecrescis erythroclada* (Kurz) Tirveng.	บักคังแดง (Bak Khang Daeng)	Nt	Tr	Wd	RT	F	Decoction	11	21	52.381	LC	TJY485
Rubiaceae	*Hymenodictyon orixense* (Roxb.) Mabb.	ส้มกบ (Som Kop)	Nt	Tr	Wd	RT	F	Decoction	6	6	100.000	LC	TJY570
Rubiaceae	*Morinda elliptica* (Hook.f.) Ridl.	ยอป่า (Yo Pa)	Nt	Tr	Wd	LV	F	Decoction	3	8	37.500	NE	TJY555
Rubiaceae	*Oxyceros horridus* Lour.	คัดเค้า (Khat Khao)	Nt	Sh	Bt	SM	F	Decoction	5	13	38.462	NE	TJY569
Rubiaceae	*Pavetta tomentosa* Roxb. ex Sm.	ข้าวสารป่า (Khao San Pa)	Nt	Sh	Wd	LV	F	Decoction	1	9	11.111	LC	TJY571
Rubiaceae	*Psychotria monticola* Kurz	ยอเทศ (Yo Thet)	Nt	Sh	Ct	LV	F	Decoction	4	4	100.000	NE	TJY518
Rutaceae	*Aegle marmelos* (L.) Corrêa	มะตูม (Matum)	It	Tr	Ct	FT	D	Decoction	5	13	38.462	NT	TJY495
Rutaceae	*Citrus hystrix* DC.	บักกูด (Bak Kut)	Nt	Tr	Ct	LV	D	Decoction	2	14	14.286	LC	TJY0121, TJY228
Rutaceae	*Citrus* × *aurantiifolia* (Christm.) Swingle	บักนาว (Bak Nao)	It	Sh	Ct	LV	F	Decoction	5	14	35.714	NE	TJY0119, TJY229
Rutaceae	*Clausena excavata* Burm.f.	สมัดใหญ่ (Samat Yai)	Nt	Sh	Wd	SM	F	Decoction	4	11	36.364	LC	TJY496
Rutaceae	*Clausena harmandiana* (Pierre) Guillaumin	ส่องฟ้าดง (Song Fa Dong)	Nt	Sh	Wd	RT	F	Decoction	8	15	53.333	LC	TJY521
Rutaceae	*Harrisonia perforata* (Blanco) Merr.	คนทา (Khontha)	Nt	Sh	Wd	RT	D	Decoction	7	11	63.636	LC	TJY499
Rutaceae	*Micromelum glanduliferum* B.Hansen	หัสคุณ (Hatsakhun)	Nt	Sh	Wd	LV	F	Decoction	5	5	100.000	NE	TJY516
Rutaceae	*Micromelum minutum* (G.Forst.) Wight & Arn.	สมัดน้อย (Samat Noi)	Nt	Sh	Wd	SM	F	Decoction	3	12	25.000	LC	TJY388
Rutaceae	*Naringi crenulata* (Roxb.) Nicolson	กระแจะ (Krachae)	Nt	Tr	Ct	BK	F	Decoction	6	16	37.500	LC	TJY517
Santalaceae	*Santalum album* L.	จันทน์ขาว (Chan Khao)	It	Tr	Ct	SM	F	Decoction	5	13	38.462	VU	TJY497
Santalaceae	*Scleropyrum pentandrum* (Dennst.) Mabb.	นมวัว (Nom Wua)	Nt	Tr	Wd	SM	F	Decoction	3	11	27.273	LC	TJY523
Sapindaceae	*Lepisanthes rubiginosa* (Roxb.) Leenh.	บักหวดข่า (Bak Uat Kha)	Nt	Tr	Bt	RT	F	Decoction	2	14	14.286	LC	TJ-F0043
Sapotaceae	*Mimusops elengi* L.	พิกุล (Phikun)	Nt	Tr	Ct	IF	D	Decoction	7	17	41.176	LC	TJY502
Simaroubaceae	*Brucea javanica* (L.) Merr.	ราชดัด (Ratchadat)	Nt	Sh	Wd	RT	F	Decoction	2	11	18.182	LC	TJY574
Simaroubaceae	*Eurycoma longifolia* Jack	ปลาไหลเผือก (Pla Lai Phueak)	Nt	Sh	Ct	RT	F	Decoction	5	14	35.714	LC	TJY536
Smilacaceae	*Smilax corbularia* Kunth	ข้าวเย็นเหนือ (Khao Yen Nuea)	Nt	Cl	Ct	RZ	D	Decoction	5	14	35.714	NE	TJY504
Symplocaceae	*Symplocos racemosa* Roxb.	เหมือดลอด (Mueat Lot)	Nt	Tr	Wd	SM	F	Decoction	9	9	100.000	LC	TJY687
Vitaceae	*Ampelocissus martini* Planch.	บักโก่ย (Bak Koi)	Nt	Cl	Wd	LV	F	Decoction	10	14	71.429	NE	TJY0050, TJY370, TJ-F0026
Vitaceae	*Cissus hastata* Miq.	เถาคันขาว (Thao Khan Khao)	Nt	Cl	Wd	RT	F	Decoction	8	11	72.727	NE	TJY713
Vitaceae	*Leea rubra* Blume	กะตังใบแดง (Katang Bai Daeng)	Nt	Sh	Wd	RT	F	Decoction	3	8	37.500	NE	TJY515
Zingiberaceae	*Alpinia galanga* (L.) Willd.	ข่า (Kha)	Nt	Hb	Ct	RZ	F	Decoction	7	14	50.000	NE	TJY0040, TJY211
Zingiberaceae	*Alpinia zerumbet* (Pers.) B.L.Burtt & R.M.Sm.	ข่าคม (Kha Khom)	Nt	Hb	Ct	RZ	F	Decoction	8	8	100.000	DD	TJY514
Zingiberaceae	*Curcuma zedoaria* (Christm.) Roscoe	ขมิ้นอ้อย (Khamin Oi)	It	Hb	Ct	RZ	F	Eaten fresh.	6	11	54.545	DD	TJY527
Zingiberaceae	*Kaempferia galanga* L.	เปราะหอม (Pro Hom)	Nt	Hb	Ct	WP	F	Decoction	1	7	14.286	DD	TJY537
Zingiberaceae	*Kaempferia marginata* Carey ex Roscoe	ตูบหมูบ (Tup Mup)	Nt	Hb	Wd	WP	F	Decoction	5	17	29.412	NE	TJY0047, TJY420

Abbreviations: Distribution in Thailand: It (introduced); Nt (native). Habit (H): Cl (climber); Hb (herb); Sh (shrub); Tr (tree). Resource (RS): Bt (both cultivated and wild); Ct (cultivated); Wd (wild). Used parts: BK (bark); FT (fruit); IF (inflorescence); LV (leaf); RT (root); RZ (rhizome); SE (seed); SM (shoot); TU (tuber); WP (whole plant). Condition of plant material (CoP): D (dry); F (fresh). Conservation status (IUCN): data deficient (DD); endangered (EN); least concern (LC); near threatened (NT); not evaluated (NE); vulnerable (VU). Voucher number (VN).

**Table 2:** Demographic characteristics and geographic distribution of informants, including district, village, GPS coordinates, gender, ethnicity, language, and religion.

District	Village Name	GPS Coordinates		Gender		Ethnicity	Language	Religion
		Latitude (N, S)	Longitude (E, W)	Male	Female			
Kham Khuean Kaeo	Bung Wai	15°39′46.76″ N	104°13′31.68″ E	2	2	Lao Isan	Isan language	Theravāda Buddhism
	Na Kae	15°35′51.45″ N	104°25′34.28″ E	2	2	Lao Isan	Isan language	Theravāda Buddhism
	Nong Kob	15°40′59.53″ N	104°22′08.12″ E	2	2	Lao Isan	Isan language	Theravāda Buddhism
Kho Wang	Mak Mai	15°21′00.10″ N	104°21′18.07″ E	2	2	Lao Isan	Isan language	Christianity, Theravāda Buddhism
	Phon Baeng	15°23′24.55″ N	104°16′09.57″ E	2	2	Lao Isan	Isan language	Christianity, Theravāda Buddhism
	Tum	15°25′15.41″ N	104°20′26.46″ E	2	2	Lao Isan	Isan language	Christianity, Theravāda Buddhism
Kut Chum	Kam Maet	15°55′12.20″ N	104°21′07.23″ E	2	2	Lao Isan	Isan language	Theravāda Buddhism
	Nong Ruea	16°04′49.05″ N	104°19′51.92″ E	2	2	Lao Isan	Isan language	Theravāda Buddhism
	Sang Tae	16°06′42.24″ N	104°09′39.97″ E	2	2	Lao Isan	Isan language	Theravāda Buddhism
Loeng Nok Tha	Bung Kha	16°08′33.38″ N	104°39′03.76″ E	2	2	Lao Isan	Isan language	Theravāda Buddhism
	Na Chan	16°09′18.18″ N	104°32′54.30″ E	2	2	Lao Isan	Isan language	Theravāda Buddhism
	Phong	16°15′36.25″ N	104°19′20.43″ E	2	2	Lao Isan	Isan language	Theravāda Buddhism
Maha Chana Chai	Hua Dong	15°33′15.30″ N	104°09′32.58″ E	2	2	Lao Isan	Isan language	Theravāda Buddhism
	Mueat Pa Tong	15°29′02.85″ N	104°15′30.40″ E	2	2	Lao Isan	Isan language	Theravāda Buddhism
	Yang Noi	15°31′57.38″ N	104°20′43.67″ E	2	2	Lao Isan	Isan language	Theravāda Buddhism
Mueang	Du Thung	15°53′10.87″ N	104°04′46.62″ E	2	2	Lao Isan	Isan language	Theravāda Buddhism
	Khilek	15°48′49.11″ N	104°11′04.54″ E	2	2	Lao Isan	Isan language	Theravāda Buddhism
	Nong Pet	15°52′01.66″ N	104°19′48.36″ E	2	2	Lao Isan	Isan language	Theravāda Buddhism
Pa Tio	Khok Sa-at	15°53′49.60″ N	104°25′22.64″ E	2	2	Lao Isan	Isan language	Theravāda Buddhism
	Nong Chum	15°57′20.18″ N	104°29′15.63″ E	2	2	Lao Isan	Isan language	Theravāda Buddhism
	Tao Hai	15°45′14.70″ N	104°21′59.38″ E	2	2	Lao Isan	Isan language	Theravāda Buddhism
Sai Mun	Kham Phai	16°00′12.47″ N	104°08′41.49″ E	2	2	Lao Isan	Isan language	Theravāda Buddhism
	Khok Kong	15°59′38.89″ N	104°10′00.56″ E	2	2	Lao Isan	Isan language	Theravāda Buddhism
	Kut Kwang	16°00′48.59″ N	104°07′37.80″ E	2	2	Lao Isan	Isan language	Theravāda Buddhism
Thai Charoen	Khu Noi	15°59′24.01″ N	104°29′51.26″ E	2	2	Lao Isan	Isan language	Christianity, Theravāda Buddhism
	Lao Han Sai	16°11′32.87″ N	104°23′58.07″ E	2	2	Lao Isan	Isan language	Christianity, Theravāda Buddhism
	Non Daeng	16°09′25.77″ N	104°24′38.69″ E	2	2	Lao Isan	Isan language	Christianity, Theravāda Buddhism

## Data Availability

The data presented in this study are available upon request from the first and corresponding author.

## Supplementary Materials

The following supporting information can be downloaded at https://www.mdpi.com/article/10.3390/plants15152373/s1, Table S1: Fidelity level (FL), informant use (I_u_), and informant citations (I_p_) of antipyretic medicinal plants documented in Yasothon Province, northeastern Thailand, ranked in descending order of FL and I_u_.
