# Supplementary material for: Traditional Antipyretic Medicinal Plants Used in Yasothon Province, Northeastern Thailand
Source: Plants (Basel). 2026 Aug 2;15(15):2373. doi: 10.3390/plants15152373 (PMC13468512; doi:10.3390/plants15152373)
Supplement: Supplementary file 1 [file plants-15-02373-s001.zip › plants-4408505-supplementary.pdf]

plants

MDPI

Supplementary

Traditional Antipyretic Medicinal Plants Used in Yasothon Province, Northeastern Thailand

Table S1. Fidelity level (FL), informant use (I_u), and informant citations (I_p) of antipyretic medicinal plants documented in Yasothon Province, northeastern Thailand, ranked in descending order of FL and I_u.

Scientific name	I_p	I_u	FL
Symplocos racemosa Roxb.	9	9	100.000
Alpinia zerumbet (Pers.) B.L.Burtt & R.M.Sm.	8	8	100.000
Mallotus philippensis (Lam.) Müll.Arg.	8	8	100.000
Myxopyrum smilacifolium subsp. *confertum* (Kerr) P.S.Green	8	8	100.000
Gnetum montanum Markgr.	7	7	100.000
Pterospermum lanceifolium Roxb. ex DC.	7	7	100.000
Antidesma sootepense Craib	6	6	100.000
Combretum pilosum Roxb. ex G.Don	6	6	100.000
Diospyros ferrea (Willd.) Bakh.	6	6	100.000
Gnetum macrostachyum Hook.f.	6	6	100.000
Hymenodictyon orixense (Roxb.) Mabb.	6	6	100.000
Dracaena angustifolia (Medik.) Roxb.	5	5	100.000
Imbralyx leucanthus var. *leucanthus*	5	5	100.000
Micromelum glanduliferum B.Hansen	5	5	100.000
Strophioblachia fimbricalyx Boerl.	5	5	100.000
Anthoshorea roxburghii (G.Don) P.S.Ashton & J.Heck.	4	4	100.000
Cladogynos orientalis Zipp. ex Span.	4	4	100.000
Diospyros undulata Wall. ex G.Don	4	4	100.000
Psychotria monticola Kurz	4	4	100.000
Sterculia lanceolata Cav.	4	4	100.000
Crypteronia paniculata Blume	3	3	100.000
Memecylon grande Retz.	3	3	100.000
Mitrephora maingayi Hook.f. & Thomson	3	3	100.000
Plumbago zeylanica L.	3	3	100.000
Sterculia guttata Roxb.	2	2	100.000
Cissus hastata Miq.	8	11	72.727
Ampelocissus martini Planch.	10	14	71.429
Barleria strigosa Willd.	7	10	70.000
Diospyros ehretioides Wall. ex G.Don	6	9	66.667
Pandanus odorifer (Forssk.) Kuntze	6	9	66.667
Harrisonia perforata (Blanco) Merr.	7	11	63.636

Eulophia attenuata (Griff.) M.W.Chase, Kumar & Schuit.	5	8	62.500
Areca catechu L.	6	10	60.000
Calotropis gigantea (L.) W.T.Aiton	6	10	60.000
Catunaregam tomentosa (Blume ex DC.) Tirveng.	8	14	57.143
Stephanotis volubilis (L.f.) S.Reuss, Liede & Meve	8	14	57.143
Ardisia helferiana Kurz	4	7	57.143
Dalbergia cultrata T.S.Ralph	4	7	57.143
Sindora siamensis Teijsm. ex Miq.	5	9	55.556
Bridelia harmandii Gagnep.	6	11	54.545
Cassia fistula L.	6	11	54.545
Curcuma zedoaria (Christm.) Roscoe	6	11	54.545
Cymbopogon citratus (DC.) Stapf	6	11	54.545
Diospyros filipendula Pierre ex Lecomte	7	13	53.846
Phyllodium longipes (Craib) Schindl.	7	13	53.846
Clausena harmandiana (Pierre) Guillaumin	8	15	53.333
Dioecrescis erythroclada (Kurz) Tirveng.	11	21	52.381
Alpinia galanga (L.) Willd.	7	14	50.000
Ficus racemosa L.	6	12	50.000
Tiliacora triandra (Colebr.) Diels	6	12	50.000
Aporosa villosa (Lindl.) Baill.	5	10	50.000
Blumea balsamifera (L.) DC.	5	10	50.000
Morus alba L.	5	10	50.000
Phyllodium pulchellum (L.) Desv.	5	10	50.000
Dillenia ovata Wall. ex Hook.f. & Thomson	6	13	46.154
Polyalthia debilis (Pierre) Finet & Gagnep.	6	13	46.154
Cinnamomum verum J.Presl	6	14	42.857
Cyanthillium cinereum (L.) H.Rob.	6	14	42.857
Embelia ribes Burm.f.	3	7	42.857
Mansonia gagei J.R.Drumm.	3	7	42.857
Terminalia chebula Retz.	3	7	42.857
Diospyros decandra Lour.	5	12	41.667
Uvaria rufa Blume	5	12	41.667
Mimusops elengi L.	7	17	41.176
Tinospora crispa (L.) Hook.f. & Thomson	7	17	41.176
Breynia glauca Craib	4	10	40.000
Tacca leontopetaloides (L.) Kuntze	4	10	40.000
Acalypha spiciflora Burm.f.	2	5	40.000
Clerodendrum schmidtii C.B.Clarke	2	5	40.000
Aegle marmelos (L.) Corrêa	5	13	38.462
Oxyceros horridus Lour.	5	13	38.462
Santalum album L.	5	13	38.462

Chrysanthemum indicum L.	6	16	37.500
Ficus hispida L.f.	6	16	37.500
Naringi crenulata (Roxb.) Nicolson	6	16	37.500
Leea rubra Blume	3	8	37.500
Morinda elliptica (Hook.f.) Ridl.	3	8	37.500
Clausena excavata Burm.f.	4	11	36.364
Citrus × aurantiifolia (Christm.) Swingle	5	14	35.714
Eurycoma longifolia Jack	5	14	35.714
Helicteres hirsuta Lour.	5	14	35.714
Smilax corbularia Kunth	5	14	35.714
Sida rhombifolia L.	6	17	35.294
Crotalaria pallida Aiton	7	21	33.333
Tabernaemontana bufalina Lour.	4	12	33.333
Croton crassifolius Geiseler	3	9	33.333
Xylopia vielana Pierre	2	6	33.333
Crateva magna (Lour.) DC.	4	13	30.769
Phyllanthus collinsiae Craib	4	13	30.769
Tamarindus indica L.	4	13	30.769
Sophora exigua Craib	3	10	30.000
Kaempferia marginata Carey ex Roscoe	5	17	29.412
Mangifera indica L.	6	21	28.571
Crateva religiosa G.Forst.	4	14	28.571
Senna tora (L.) Roxb.	4	14	28.571
Canthium berberidifolium E.T.Geddes	5	18	27.778
Hibiscus sabdariffa L.	5	18	27.778
Phanera strychnifolia (Craib) K.W.Jiang, S.R.Gu & T.Y.Tu	5	18	27.778
Azadirachta indica A.Juss.	3	11	27.273
Beaumontia murtonii Craib	3	11	27.273
Scleropyrum pentandrum (Dennst.) Mabb.	3	11	27.273
Xylia xylocarpa var. *kerrii* (Craib & Hutch.) I.C.Nielsen	3	11	27.273
Bixa orellana L.	3	12	25.000
Cananga brandisiana (Pierre) Saff.	3	12	25.000
Micromelum minutum (G.Forst.) Wight & Arn.	3	12	25.000
Dillenia hookeri Pierre	2	8	25.000
Ellipanthus tomentosus Kurz	2	8	25.000
Carthamus tinctorius L.	4	17	23.529
Goniothalamus laoticus (Finet & Gagnep.) Bân	4	17	23.529
Memecylon edule Roxb.	3	13	23.077
Annona squamosa L.	4	18	22.222
Alyxia schlechteri H.Lév.	2	9	22.222
Afzelia xylocarpa (Kurz) Craib	4	20	20.000

Senna occidentalis (L.) Link	2	10	20.000
Clitoria ternatea L.	4	22	18.182
Brucea javanica (L.) Merr.	2	11	18.182
Flemingia stricta Roxb.	2	11	18.182
Uvaria hirsuta Jack	3	18	16.667
Crinum asiaticum L.	2	12	16.667
Irvingia malayana Oliv. ex A.W.Benn.	4	25	16.000
Uvaria siamensis (Scheff.) L.L.Zhou, Y.C.F.Su & R.M.K.Saunders	3	19	15.789
Capparis micracantha DC.	2	14	14.286
Citrus hystrix DC.	2	14	14.286
Lepisanthes rubiginosa (Roxb.) Leenh.	2	14	14.286
Kaempferia galanga L.	1	7	14.286
Strychnos nux-blanda A.W.Hill	2	16	12.500
Croton persimilis Müll.Arg.	1	8	12.500
Pavetta tomentosa Roxb. ex Sm.	1	9	11.111
Phyllanthus emblica L.	1	20	5.000
